# Longitudinal domain wall formation in elongated assemblies of ferromagnetic nanoparticles

**DOI:** 10.1038/srep14536

**Published:** 2015-09-29

**Authors:** Miriam Varón, Marco Beleggia, Jelena Jordanovic, Jakob Schiøtz, Takeshi Kasama, Victor F. Puntes, Cathrine Frandsen

**Affiliations:** 1 Technical University of Denmark, Department of Physics, 2800 Kgs. Lyngby, Denmark; 2 Institut Català de Nanotecnologia, Campus UAB, 08193 Barcelona, Spain; 3 Technical University of Denmark, Center for Electron Nanoscopy, 2800 Kgs. Lyngby, Denmark; 4 Helmholtz-Zentrum-Berlin fuer Materialen und Energie, Hahn-Meitner-Platz 1, 14109 Berlin, Germany; 5 Danish National Research Foundation Center for Individual Nanoparticle Functionality, Technical University of Denmark, 2800 Kgs. Lyngby, Denmark; 6 Vall d’Hebron Institut de Recerca (VHIR), 08035, Barcelona, Spain; 7 Institut Català de Recerca i Estudis Avançats (ICREA), 08010 Barcelona, Spain

## Abstract

Through evaporation of dense colloids of ferromagnetic ~13 nm ε-Co particles onto carbon substrates, anisotropic magnetic dipolar interactions can support formation of elongated particle structures with aggregate thicknesses of 100–400 nm and lengths of up to some hundred microns. Lorenz microscopy and electron holography reveal collective magnetic ordering in these structures. However, in contrast to continuous ferromagnetic thin films of comparable dimensions, domain walls appear preferentially as longitudinal, i.e., oriented parallel to the long axis of the nanoparticle assemblies. We explain this unusual domain structure as the result of dipolar interactions and shape anisotropy, in the absence of inter-particle exchange coupling.

One of the few practical avenues for creating superstructures of nanoparticles (NPs) is self-assembly (SA). The process can be considered akin to crystallization, where elements rearrange themselves into a more or less periodic crystal structure. Under appropriate conditions, colloidal NPs can self-assemble into structures with long-range periodic order^1^,^2^. Moreover, spontaneous assembly of monodisperse ligand-capped NPs may occur during drying^1^,^3^,^4^,^5^,^6^, where evaporation of the solvent from colloidal particle dispersion allows self-organization of the particles on top of a desired substrate^7^,^8^. During this process, the solvent, the drying kinetics, substrate roughness, solvent wetting/dewetting, hydrodynamic effects, and self-diffusion of the NPs on the substrate, play important roles and can lead to unusual non-equilibrium structures, e.g. dendritic structures^9^,^10^. In cases of magnetic NP assembly, magnetic dipolar interactions often become increasingly important during drying^11^ where particle kinetics ceases, and this can lead to formation of anisotropic structures such as wires, chains^12^,^13^,^14^ and rings^15^. Moreover, dipolar interactions between single-domain ferro- or ferrimagnetic NPs may be strong and lead to collective magnetic ordering in NP assemblies^16^,^17^,^18^,^19^,^20^.

A major physics question related to magnetic assemblies of NPs is which magnetic order, i.e. which domain structure, and which magnetic properties may be associated with these structures, but few techniques have the ability to reveal this at the scale of the structures. Magnetic domains that occur over several particles due to collective magnetic ordering in NP assemblies may at first be expected to be similar to magnetic domains in more conventional materials, with the only difference being that atomic spins are replaced by the magnetic moments of the single-domain NPs. However, this is not necessarily the case. In traditional continuous magnetic materials, properties and domains structures are largely determined by the intrinsic exchange coupling, but in magnetic NP assemblies, where particles contain a surfactant layer, inter-particle exchange interactions are negligible, and hence different properties may emerge depending on particle types and particle arrangements^16^,^17^,^18^,^19^. As such, magnetic NP assemblies represent distinct materials, which deserve interest, both in terms of physical understanding, and in terms of exploitation of potential applications, e.g. in magnetic devices that can be designed bottom-up.

Here we report fabrication of extensive, anisotropic, “rope-like” NP structures by drying-mediated aggregation of ligand-capped ferromagnetic ~13 nm ε-Co particles onto a carbon-based substrate. We show by use of transmission electron microscopy (TEM) based techniques for magnetic imaging^21^ that collective magnetic ordering and unconventional domain structures (i.e., longitudinal walls) exist in these structures. The domain structures are deemed “unconventional” because, unlike conventional/continuous materials, there is no exchange energy cost proportional to the cross-sectional area of a domain wall, thus walls can be very long in dipolar-coupled magnetic materials; they can also feature a 180-degree magnetization rotation between neighbouring particle chains corresponding to zero wall width. We provide a theoretical justification for the longitudinal domain wall formation based on numerical simulations.

## Experimental Methods and Results

### Sample fabrication

Dense colloids of fairly monodisperse ε-Co NPs with diameters of approx. 13 nm (12.5 ± 1.1 nm) were synthesized in an Ar atmosphere by thermal decomposition of the Co_2_(CO)_8_ precursor in a boiling (181 °C) mixture of organic solvent (1,2-dichlorobenzene anhydrous) and surfactants (trioctylphosphine oxide and oleic acid)^3^,^22^.

Nanoparticle assemblies were achieved by depositing drops of the colloidal solution onto amorphous carbon on standard 400-mesh copper TEM grids from Ted Pella INC and letting the colloid dry in oxygen-free conditions inside a glove box. During the drying process the samples were covered by a Petri dish in order to slow down evaporation of dichlorobenzene and allow more time for the particles to arrange during drying.

### Bright field TEM

**Figure 1:**
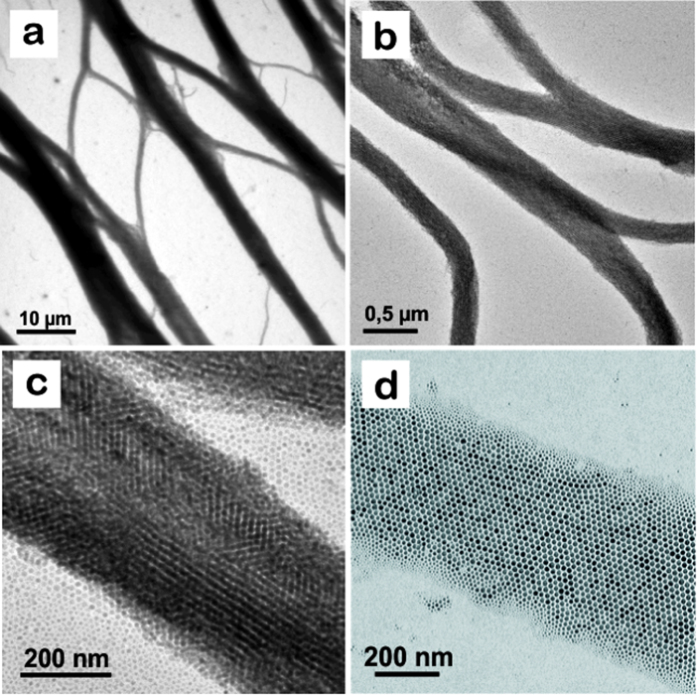
TEM images of elongated structures (“ropes”) of 13 nm ε-Co particles formed by solvent evaporation of colloids on amorphous carbon. (**a–c**) NP multilayers. (**d**) NP monolayer.

**Figure 2:**
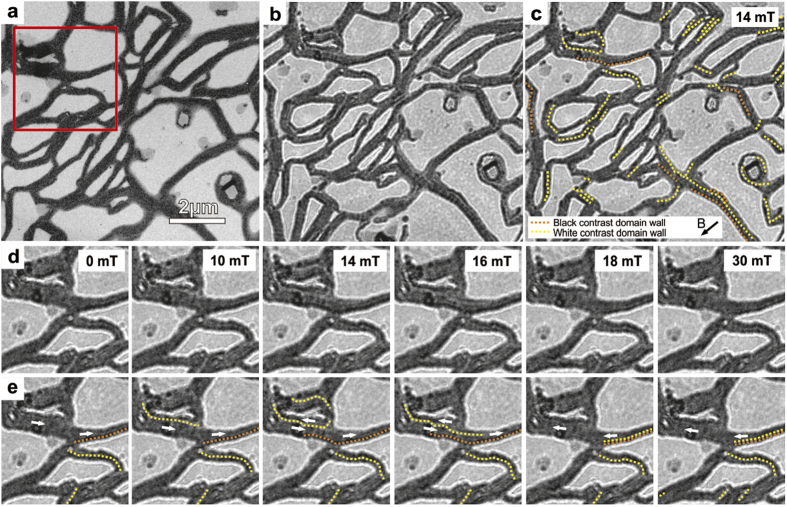
(**a**) TEM bright field images of elongated NPs structures. (**b**) Lorentz microscope image of elongated NPs structures at under-focus conditions and in an applied field of 14 mT (= an in-plane field of 7 mT). (**c**) the most distinct domain walls in image b highlighted by dotted lines (yellow = bright contrast domain walls, orange = dark contrast domain walls). The in-plane field direction is given by the arrow on the inset. (**d**) Lorentz microscope images of the region indicated by a red square in image a, showing the evolution of the domain walls in increasing applied field (see also Movie 1 in Supplementary Information). (**e**) the most distinct domain walls in image d highlighted by dotted lines. Local magnetization directions are also indicated by white arrows. We emphasize that not all domain walls are highlighted. Especially, dark domain wall contrast is hard to distinguish; hence light (yellow) walls appear overrepresented and often adjacent although in-between dark (orange) walls are expected.

Figure 1 shows bright field TEM images of the NP assemblies on amorphous carbon (obtained by an FEI Tecnai T20 G2 TEM operated at 200 kV, Fig. 1a–c, and an FEI Titan 80–300ST TEM operated at 120 kV, Fig. 1d). It can be seen that the applied drop deposition followed by solvent evaporation has lead to the formation of very extensive, almost rope- (or net-) like structures of NPs with thicknesses up to around 400 nm and lengths up to hundreds of micrometers. These structures have a somewhat “organic” shape (see also Fig. 2a) and bear some resemblance to the loop structures observed by Ku *et al.* in their studies of evaporating magnetic NP solutions onto a similar amorphous carbon substrate^10^. The assemblies consist of mono- or multilayers of NPs. The average thickness of the ropes depends on different factors such as particle concentration and amount of suspension per TEM grid, but the thicknesses of the individual ropes that are achieved during the drying process are beyond our control and vary over the grid (see also Fig. S4). From image contrast (see Supplementary Information), we estimate that most of the ropes in Fig. 2a are monolayers. High magnification TEM images show that the Co NPs are mostly hexagonally packed (Fig. 1c,d), with the particle lattice planes being parallel to and at 60° to the long axis of the structures.

### Lorentz microscopy

We applied Lorentz electron microscopy to address magnetic ordering, in particular areas of different particle magnetization directions (domains) and in-field domain behavior in the ropes. Lorentz microscopy captures the Lorentz deflection of electrons traveling within a magnetic field, and it has been used extensively to reveal magnetic domain structures in thin ferromagnetic films. Here we applied the Fresnel mode of Lorentz microscopy^23^, where the working principle is that electrons are deflected uniformly in each domain, resulting in the beam converging/diverging below the walls in the sample. Then, by taking the image out of focus, i.e. in a plane not coincident by the sample plane, contrast is generated at the domain boundaries (delineated by bright and dark “fringes”). Magnetic contrast is also generated at the edges of the rope, where the magnetization drops to zero. For example, the magnetic deflection of electrons by a uniformly magnetized rope will add a contrast asymmetry between opposing edges. This contrast superimposes with the electrostatic contrast, which is usually much stronger.

The ropes were initially magnetized in one direction in the TEM by applying a large magnetic field (>1 T) in the direction of the electron beam using the objective lens and with the sample tilted 30° (*i.e.*, the angle between sample plane and field is 60° and the field in the plane of the sample was ≥0.5 T). Subsequently, while maintaining the sample tilt, the objective lens was used to obtain reverse field magnetizations while imaging. Starting from zero-field, the value of the objective lens was varied, in steps of 2 mT, until it reached a reverse field value of 40 mT. Fresnel images acquired during magnetization reversal are shown in Fig. 2b–e.

From the Lorentz images in Fig. 2b–e, it is seen that many ropes show lines of contrast (dark/bright lines) within their structure and along their length. The line contrast suggests that the magnetic NPs show collective magnetic ordering with domain structures. In this case, the ropes without contrast represent ropes with dipolar ferromagnetic order (the magnetic moments are aligned), while ropes with contrast lines appear to have domain walls that are longitudinal i.e. parallel to the length of the ropes (the magnetic moments change their alignment directions at the domain wall). In Fig. 2c,e, we have attempted to highlight with dotted yellow and orange lines the most apparent lines of bright and dark domain wall contrast, respectively. The domain contrast is sometimes difficult to distinguish and not all lines of contrast representing domain walls may have been highlighted. Especially, the dark lines of contrast (marked orange in Fig. 2c,e) are hard to resolve. Also, some domain walls may produce less distinct contrast than others (sharp longitudinal 180° domain walls being most pronounced) and therefore it is possible to find longitudinal domain walls that appear to terminate in the middle of the ropes. However, the longitudinal domain walls are in many cases clearly distinguishable, especially as they sweep over the structures in the transverse direction of the ropes, typically within fields of 5–30 mT (see Figs 2e, S3 and Movie 1 in the Supplementary Information). Movie 1 in Supplementary Information shows domain wall evolution with field in steps of 2 mT and we emphasize that it is particularly helpful in resolving the domain wall nucleation and propagation. In many of the ropes we observed domain wall nucleation with reverse fields around 20 mT (examples in Fig. S3b,c). We notice that there is a tendency that domain walls appear less prevalent in thin long ropes (e.g. those ropes pointing downwards from the lower right corner of the red square in Fig. 2a).

### Electron Holography

From measurements by off-axis electron holography, whereby the remanent magnetic ordering could be resolved with almost single particle resolution, see Supplementary Information, we confirm that the magnetic order in the NP assemblies is collective with the length of the particle assemblies as the preferred axis of magnetization (Fig. S1). Moreover, electron holography shows that domains structures may exist in the assemblies with domain walls and domain magnetizations preferentially in the length direction (Fig. S2).

## Magnetic Simulations and Discussion

For comparison, elongated continuous strips of Co with comparable dimensions, e.g. nanowires made by focused electron beam induced deposition^24^, do not support longitudinal domain walls. Instead, transversal walls (or vortex walls) between two head-to-head domains are common in ferromagnetic thin wires^24^,^25^, because these short walls have the lowest possible exchange cost. Our observations of longitudinal domain walls in elongated NP assemblies stress the difference between magnetic nanoparticle materials and continuous materials.

In order to understand how a longitudinal domain wall is stabilized in an elongated nanoparticle structure, we first note that, in general, the absence of inter-particle exchange coupling introduces a magnetic “softness”: There is no exchange energy cost of the domain wall itself and the energy is solely determined by the interactions between the magnetic dipoles. It may therefore appear favorable to introduce longitudinal domain walls in elongated structures as that keeps the magnetic field inside the structure and hence lowers the global magnetostatic energy. On the other hand, there is a dipolar energy cost in forming a domain wall. Due to the spatial anisotropy of the dipolar interaction, this cost depends on the particle arrangements: In a simple square lattice of magnetic moments, the favored magnetic structure is “ferromagnetically” ordered chains along the dipole direction, with adjacent chains being “antiferromagnetically” ordered (*i.e.,* 180° walls between adjacent chains are favored). However, in a hexagonal (close-packed) lattice, the nearest neighbor coupling is always ferromagnetic, and consequently ferromagnetic ordering is favored locally^26^,^27^.

With the purpose of elucidating the magnetic domain ordering in elongated nanoparticle assemblies, we simulated closed-packed 2D monolayer assemblies of magnetic NPs with width W = 2, 4, 6, ..., 24 particles and length L = 2, 4, 6, …, 140 particles. A magnetic dipole moment is assigned to each particle. For each assembly, three initial magnetic dipole configurations were investigated: 1) a single-domain ferromagnetic state (denoted the F-state) where all magnetic dipole moments are aligned parallel to the length of the assembly, 2) a T-state with a single transversal domain wall (i.e., a domain wall perpendicular to the length of the assembly and the two domains “head-to-head”), and 3) an L-state with a single longitudinal domain wall (i.e. a domain wall parallel to the assembly length, with two narrow domains on each side). The domain wall in the T- and L-states is placed symmetrically to yield zero net moment.

**Figure 3:**
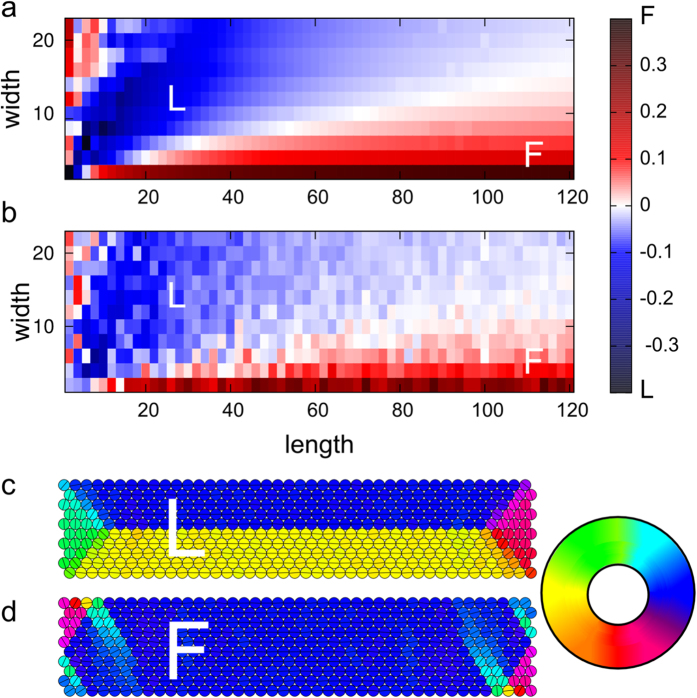
Calculated diagrams of the most favorable domain state for (**a**) ordered and (**b**) disordered NP structures showing the unitless dipolar energy difference between the relaxed F- and L-state per particle, scaled by the factor μ_0_μ^2^/4πr^3^, with μ being the particle moment and r the (average) particle distance. Red shows where the F-phase is preferred, blue where L-phase is preferred. Units of length and width are number of particles. (**c**,**d**) The relaxed L- and F-states for ordered NP assemblies (W = 10, L = 42) are shown in c and d, respectively. The domain wall in c has no width: adjacent particles have opposite magnetization. The color wheel denotes the magnetic moment directions in c and d.

The magnetic moments of the three states (F, T, L) were allowed to relax using damped Newtonian dynamics (see Supplementary Information and ref. 28 for details). The initial states of the elongated structures relax to somewhat similar states (i.e., we do not observe transitions between the three states during relaxation). For example, when the L-state relaxes, a long longitudinal wall remains and the change happens mainly close the ends (see example of relaxed L-state in Fig. 3c). Similarly, in case of a relaxed F-state (example in Fig. 3d) most of the moments are still aligned and mainly the moments near the ends have turned away from perfect alignment. Only for small aspect ratios L/W <~1–2, the initial states develop into more complicated domain structures that cannot be clearly identified as F-, L-, or T-type during relaxation. For each system size we determine the relaxed state with the lowest energy (summarized in Fig. 3a). From the simulation we find that the F- and L-states are favorable and that nanoparticle assemblies up to an aspect ratio L/W of ~7 favor the L-state over the F-state. The T-state is never favored.

The simulations represent simplifications of the real NP systems, which have loop-structures and typically consist of more than one monolayer of particles. However, the magnetic ordering in the simulated systems, which only rely on dipolar interactions, lean support to interpreting the observed superferromagnetic ordering and longitudinal domain walls as being a consequence of inter-particle dipolar interactions. We note that the simulations also support our observation (Fig. 2) of fully ferromagnetic order appearing most prevalent in narrow ropes. A longitudinal domain wall appears favorable in the simulations for wider particle ropes (L/W ~2–7) where the demagnetizing field of the entire assembly of dipoles is sufficient to compete with the local ferromagnetic alignment.

**Figure 4:**
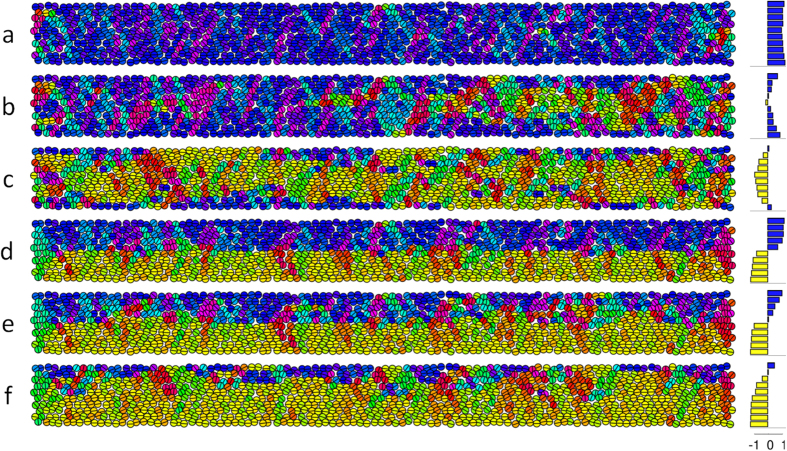
Simulation of the relaxed F-state (**a–c**) and the relaxed L-state (**d–f**) with increasing reverse fields of (**a,d**) 0 mT, (**b,e**) 35 mT, and (**c,f**) 50 mT. The colors denote the orientation of the magnetic moment (cf. the color wheel in Fig. 3). Panels on the right hand side indicate the net moment in the length direction over the widths of the assemblies.

A feature of the real NP assemblies, which we have not been included in our simulations summarized in Fig. 3a, is the disorder in the particle arrangement (i.e., the deviation from perfect close packing visible in Fig. 1d). Structural disorder may influence the magnetic ordering and we therefore investigated its’ importance. For this, we first estimated the degree of structural disorder in the experimental nanoparticle assemblies according to the methodology applied in refs 27,29, and found the disorder to be about 0.17, meaning that twice the standard deviation of the first neighbor distances is 0.17 times the mean first neighbor distance. In order to fully account for the possible effect of disorder, we introduced an even larger degree of disorder (0.27) in each of the simulated NP arrays and redid the relaxation from F-, T- and L-states. Examples of relaxed F- and an L-state with nanoparticle disorder (0.27) are shown in Fig. 4a,d, respectively. Again, we determine the relaxed states with lowest energy for each system size (results are summarized in Fig. 3b). From this we find that the disorder has very little effect on the magnetic phase diagram (Fig. 3b vs. 3a); again, only the relaxed F- and L- states are favored. The cross-over from the relaxed L-state to relaxed F-state is shifted only to a slightly higher aspect ratio of L/W ~ 8.

We further used simulations to investigate the domain wall evolution in a magnetic field applied parallel to the long axis of the NP assemblies. We started the calculations from the configuration of a disordered structure of a relaxed F- and L-state (Fig. 4a,d) and then applied a reverse magnetic field up to 50 mT in steps of 5 mT. The moments were allowed to relax in-field at each step (similar to the experimental procedure). The disorder pins the magnetization during reversal and allows us to capture it over a range of fields. This pinning during field reversal is not seen in the ordered structures. The relaxed F-state (Fig. 4a–c) shows domain nucleation at one of the ends, and in the interior, followed by expansion of the domain walls through the entire sample in a longitudinal fashion, while the domain wall moves in the transversal direction towards the edges. The relaxed L-state (Fig. 4d–f) also shows propagation of the domain wall mainly in the transversal direction. These results are similar to what we have observed experimentally (Figs 2 and S3). Moreover the simulations predict a “coercive”/de-stabilizing field of the order of 35 mT for the F- and L-states, comparable with the observations (Figs 2, S3).

## Conclusions

In conclusion, we have described the physical behaviour of self-assembled low-dimensional superstructures of magnetic nanoparticles (elongated nanoparticle assemblies of almost close-packed ~13 nm Co particles). Magnetic nanoparticle systems may in general be considered a new class of magnetic materials, since their properties may be very different from conventional magnetic materials, and can be tailored by means that are not easily accessible in other systems. We find that a fundamental difference in magnetic response emerges from the combination of exchange-decoupling between magnetic building blocks and their dipolar magnetostatic interactions. In particular, we have verified that longitudinal domain-walls can be stabilized by dipolar inter-particle interactions, in the absence of inter-particle exchange interactions. The energy of transverse/vortex walls is comparable in continuous film and nanoparticle assemblies while the energy of longitudinal walls is very large (proportional to the wall length) in continuous films and very low in nanoparticle assemblies. Therefore, while vortex walls do indeed appear in nanoparticle assemblies with comparable lateral dimensions (e.g. squares, etc.), longitudinal walls are always cheaper to form in elongated nanoparticle assemblies, but they are the most costly in continuous strips. Moreover, we find that domain wall propagation occurs in the transverse direction with reverse field for longitudinal domain walls in elongated nanoparticle assemblies and that domain structures are stabilized during reversal by imperfections and packing disorder. The domain behaviour has been captured visually by electron microscopy experiments, and the results interpreted on the basis of simulations, providing a coherent and consistent physical picture that highlights how the domain behavior of nanoparticle-materials does not involve transverse (or vortex) domain walls typically observed in thin film strips of Co of comparable dimensions.

## Additional Information

**How to cite this article**: Varón, M. *et al.* Longitudinal domain wall formation in elongated assemblies of ferromagnetic nanoparticles. *Sci. Rep.* **5**, 14536; doi: 10.1038/srep14536 (2015).

## Supplementary Material

Supplementary Information

Supplementary Movie 1
