# Supplementary material for: Longitudinal domain wall formation in elongated assemblies of ferromagnetic nanoparticles
Source: Sci Rep. 2015 Sep 29;5:14536. doi: 10.1038/srep14536 (PMC4586724; doi:10.1038/srep14536)
Supplement: Supplementary Information [file srep14536-s1.pdf]

Submitted April 22, 2015

Supplementary Information to

"Longitudinal domain wall formation in elongated assemblies of ferromagnetic nanoparticles"

authored by

Miriam Varón, Marco Beleggia, Jelena Jordanovic, Jakob Schiøtz, Takeshi Kasama, Victor F. Puntes, and Cathrine Frandsen

Electron holography

In addition to the Lorentz microscopy studies of Co NP assemblies on amorphous carbon presented in the manuscript, the magnetic ordering in the assemblies was also investigated by electron holography which allows obtaining quantitative information of the magnetic field in the sample with a spatial resolution slightly below particle size. The NPs assemblies were studied by off-axis electron holography at saturation remanence (i.e. in zero-field after application of a large magnetic field). This was done in the following way: Within the transmission electron microscope, the sample plane of the NPs assemblies on amorphous carbon (originally perpendicular to the electron beam) were tilted by ± 30°, where after the conventional microscope objective lens were turned on to apply a magnetic field of 2 T parallel to the direction of the electron beam. The objective lens was then turned off and the sample tilted back to 0° for hologram acquisition in field-free conditions and with the particles at remanence. The holograms of the different rope-like Co structures were recorded with the sample magnetized in opposite directions. For NP structures where the magnetization could be completely reversed, the non-magnetic mean inner potential contribution to the phase shift could be removed. The mean inner potential was separated from the magnetic potential as described in Ref. 1,[1] and the holographic magnetic field map created (Fig. S1b,e). For these structures we find the magnetic field $B_{\perp}$ (along the chain) of 1.36±0.35T for the monolayer and 1.33±0.34 T for the multilayer. The magnetic field was extracted from phase profile taken along the short axis of a chain, averaged over a region of 795 nm x 133 nm to decrease signal to noise. A typical chain reveals a phase shift Δφ between opposite sides of 4.27 rad for the monolayer chain and 8.68 rad for the multilayer chain (Fig. S1 c,f). To convert the phase shift into magnetic field, we use the following equation, which considers a bimodal particle size distribution:

$$\Delta\varphi = \frac{4\pi^2}{3\phi_0} B_{\perp} \left(\frac{N_1 R_1^3}{a_1} + \frac{N_2 R_2^3}{a_2} \right)$$

with R_n mean particle radii (R_1=15.4±0.7 nm, R_2=6.0±1 nm), a_n mean nearest neighbor distances (a_1=18.0±0.8 nm, a_2=11.6±2.2 nm), ϕ_0=h/2e=2.07x10^{-15} T m^2 the flux quantum, and N_n the number of 1-particle chains spanned by the profile. The formula, valid in the assumption of uniform magnetization and full alignment of all particles, takes into account the effective demagnetization factor of an infinite chain in the reduction, proportional to $2R/a$, of the total magnetic flux carried by each particle. The lower field value obtained may suggest that not necessarily the whole volume of each particle is magnetized, e.g. if there is some oxide shell around each particle.

These results indicate, in correspondence with the Lorentz microscopy observations, that the magnetic ordering of the close-packed elongated NPs structures on amorphous carbon is superferromagnetic.

For NP structures where the magnetization does not reverse, the magnetic and non-magnetic (mean inner potential) contributions to the phase shift cannot be separated with the procedure we have employed. Nevertheless, due to the sample uniformity it is possible to get some information about the general magnetic structure from the total phase image. An example is shown in Fig. S2. If we assume the mean inner potential is a constant contribution set to zero and that all the variation in the phase shift between opposite magnetized structures comes from the magnetic potential, then we can create "false" magnetic maps indicating the field directions in the ropes (see Fig. S2c and S2e). From these field maps we see that the magnetization has not been reversed. Rather, two different magnetic structures are achieved when the sample is magnetized in opposite directions (Fig S2c vs. Fig S2e). In particular, Fig. S2e shows the presence of different domains within a rope, with the domain walls preferably in the direction parallel to the length of the rope, thus confirming that a longitudinal domain wall is a stable configuration after magnetization. This supports the previous observations in Lorentz experiment at zero field after magnetization (Figure 2d).

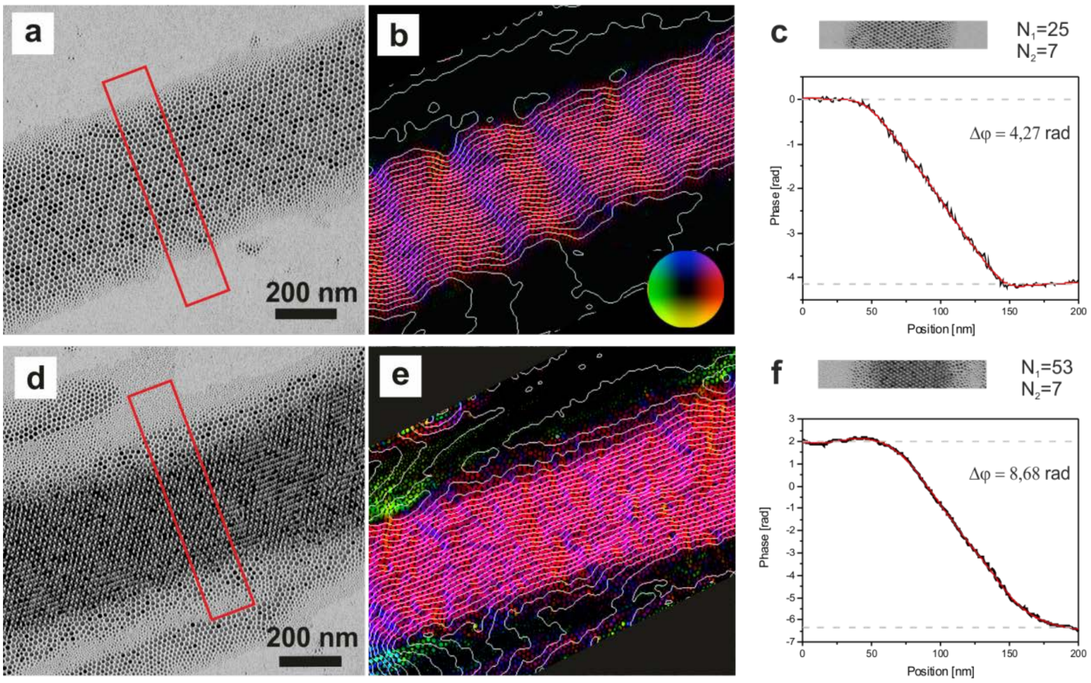

Figure S1. a,d) TEM bright field images of elongated structures of close packed monolayer (a) and multilayer (d) of particles. b,e) Holographic magnetic field maps of the same regions showed in a and d, respectively. Magnetic states after application of a field of ±2 T are shown. The white contours and the colors (described using the color wheel) provide a measure of the magnitude and direction of the projected magnetic field in the plane of the particles with a spatial resolution of ~11 nm. Each white contour represents a phase variation of 0.15 radians (b) and 0.32 radians (e). c,f) Phase profiles plotted as a function of position for the selected areas in a and d, respectively. The red lines show a smoothing of the data points. N_2 and N_2 are the approximate number of NP chains within the monolayer and the multilayer in the selected regions considering a bimodal particles size distribution.

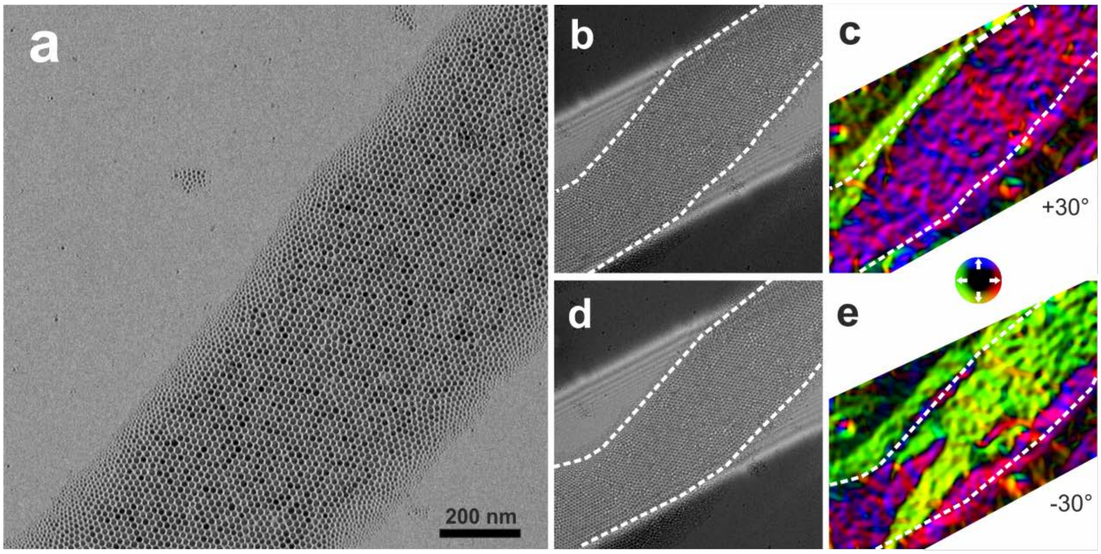

Figure S2. a) Figure S2. Close-up TEM image of a close packed monolayer of particles within an elongated structure. b,c) Electron holograms of a larger region acquired in Lorentz mode at 120 kV using a FEI Titan 80-300ST TEM. c,e) *Holographic color maps (described using the color wheel) of the reconstructed total phase image after magnetization at ±30˚.*

Lorentz microscopy

In addition to Fig. 2d, Fig. S3 shows two other regions from Fig. 2a that have also been studied in detail by Lorentz microscopy as a function of applied field (see also Movie 1 in the Supplementary Information). These structures, formed by a bilayer of NPs, also show longitudinal domain walls and domain wall propagation when increasing the applied field. The initial states of Region 2 show short longitudinal domain walls that grow in length when the field is increased above 18 mT, and eventually sweep out of the structures when the field reaches around 40 mT. Region 3 is initially free of domain walls, but shows nucleation of a short longitudinal domain wall at 24 mT; this domain wall propagates along the rope structure with increasing field until it disappears at 40 mT.

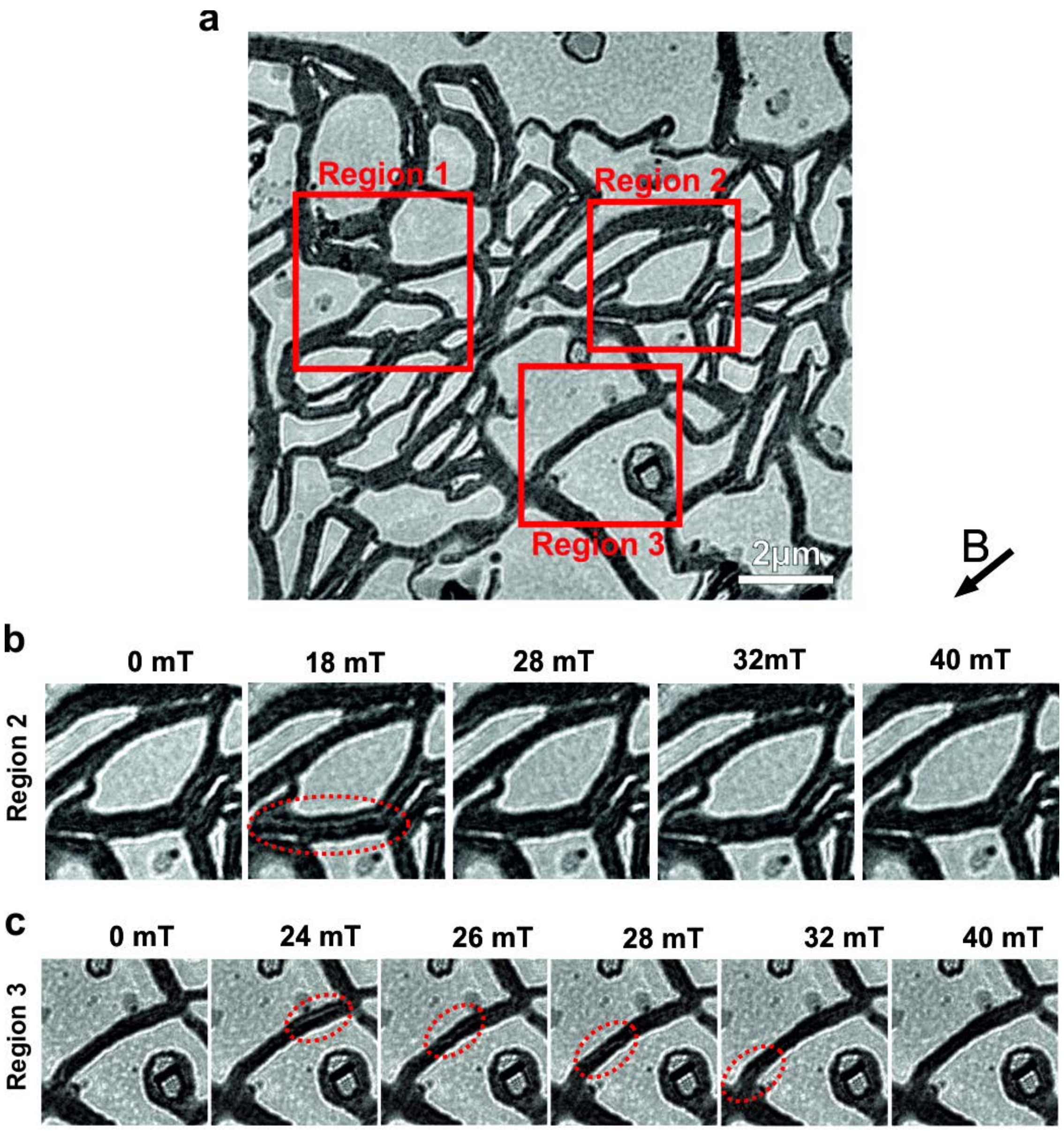

Figure S3. a) Same image as in Fig. 2a, but with region 1(Fig. 2d,e) and regions 2 and 3 indicated. b-c) Lorentz microscope images at under-focus conditions showing the evolution of the domain walls while increasing the applied reversing field from zero to 40 mT. In-plane field direction is given by the black arrow in a. b) Region 2 with an initial longitudinal domain wall, which expands at 18 mT (highlighted by a red dotted circle). c) Region 3 initially without a domain wall, showing domain wall formation and propagation between 24 and 32 mT (highlighted by red dotted circles). We emphasize that not all domain walls are highlighted.

Thickness determination

Assuming incoherent electron scattering from atoms with constant atomic number Z, the contrast $\Delta I/I$ from a change in thickness, Δt, is given as

$$\frac{\Delta I}{I} = 1 - e^{-Q\Delta t} = Q\Delta t$$

where Q is the total elastic scattering cross section.[2]

We have compared two different bright field images of the rope structures acquired at the same conditions (Figs. S4a and S4b; Fig. S4b is the same as Fig. 2a). The maximum intensity values (I) of the selected areas correspond to the intensity of the carbon film of the TEM grid, and are 1300 e^- and 1240 e^- for Figs. S4a and S4b, respectively. The normalized intensity line profiles of the two selected regions in Figs. S4a and S4b (highlighted with red square) are shown in Figs. S4c and S4d, respectively.

Figure S4c shows a decrease of intensity in three steps. The intensity decrease (*ΔI/I)* in the three steps is ~0.20, ~0.39 and ~0.52, sequentially. We have observed from zooming in the image that the first step corresponds to the presence of a monolayer. This allows us to interpret that the two next steps correspond to the presence of a double layer and a triple layer, sequentially.

Figure S4d shows an intensity change of ~0.22. By comparing this value with the ones obtained in the Figure S4c, we find that the intensity decrease corresponds to the presence of a monolayer of nanoparticles. The intensity change is almost constant in all the ropes in Figure S4b, indicating that all the ropes in the image (region of the Lorentz microscopy experiment Fig. 2a) are composed of a monolayer of nanoparticles.

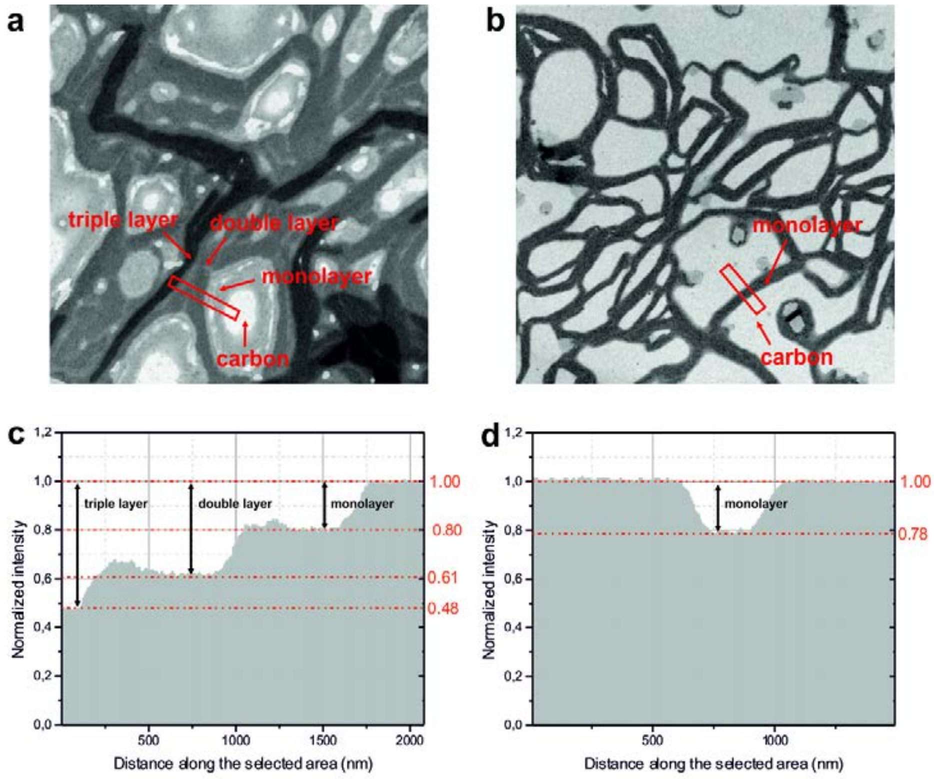

Figure S4. a) Bright field images of the sample showing rope structures with thicknesses from mono- to triple layers. b) Bright field image of the Lorentz experiment showing rope structures with thickness of monolayer. c-d) Normalized intensity profiles of the two areas indicated by a red square in images a and b, respectively.

Simulations

The model

When modelling the dynamic dipole moment in our simulations, we assume magnetically isotropic particles, where the orientation of the magnetic moment is free to rotate. As long as we are not interested in the temporal dynamics, this is mathematically equivalent to the case of highly anisotropic particles where the particle is allowed to rotate. The energy of the particles is given by

$$U = \frac{\mu_0}{4\pi} \sum_{i,j\neq i} \left(\frac{\vec{\mu}_i \cdot \vec{\mu}_j}{r_{ij}^3} - 3\frac{(\vec{\mu}_i \cdot \vec{r}_{ij})(\vec{\mu}_j \cdot \vec{r}_{ij})}{r_{ij}^5} \right) - \mu_0 \sum_i \vec{\mu}_i \cdot \vec{H}$$

where μ_0 is the vacuum permeability; $\vec{\mu}_i$ is the dipole moment of particle *i*; $\vec{r}_{ij}$ and r_{ij} are the distance vector between moment *i* and *j* and its length, respectively; and $\vec{H}$ is the applied field. As we do not allow spatial motion of the particles, we do not need a repulsion term keeping the particles apart, and the volume of the particles only enters through the size of the magnetic moment of the particle.

For the parameter values, we use values corresponding to Cobalt nanoparticles in our model, with particle diameters of 15 nm and magnetisation saturation of $M_s = 1.4\times10^6$ A/m, leading to a magnetic moment per particle of $\mu = 2.47\times10^{-18}$ A m^2, and the density of $\rho = 8.9\times10^3$ kg/m^3 leading to a moment of inertia of I = 3.54×10^{-37} kg m^2.

We use a damped molecular dynamics to find the final magnetic structures, integrating Newton's second law with a leapfrog algorithm and a Berendsen thermostat.[3,4] The timestep used was 5 ps, and the characteristic time of the Berendsen thermostat was 10 ns. The temperature was set to 100K, but the actual value used has no importance as the thermal fluctuations of nanoparticles of these sizes are negligible at any reasonable temperature. The only roles of the finite temperature are thus to allow the numerical integration to break symmetries in the initial configurations, and to prevent the system from being caught in shallow local energy minima. One simulation run consists of an equilibration phase (12000 iterations), followed by the averaging phase, where we estimate the time averaged interaction energies (28000 iterations), leading to a total simulation time of 200 ns.

Disorder of the particles

For the situation of disordered particles, we use a hexagonal grid with a grid size of r=15 nm as a starting point. The positions of the magnetic moments are moved from the grid point by a Gaussian distributed random vector. With this method some moments may lie closer to each other than σ=15 nm (assumed size of particles), but as we model point dipole moments there is no issue of overlapping particles.

REFERENCES

1. Dunin-Borkowski, R. E. Magnetic microstructure of magnetotactic bacteria by electron holography. *Science.* **282,** 1868–1870 (1998).
2. Williams, D. B. & Carter, C.B. Transmission Electron Microscopy. 729 (Plenum Press, New York, 1996)
3. Allen, M. P. & Tildesley, D. J. *Computer Simulation of Liquids, Oxford Science Publications*. 1-408 (Oxford University Press, New York, 1989).
4. Andersen, H. C. Molecular dynamics simulations at constant pressure and/or temperature. *J. Chem. Phys.* **72,** 2384 (1980).
